# Identification of Interactions in the NMD Complex Using Proximity-Dependent Biotinylation (BioID)

**DOI:** 10.1371/journal.pone.0150239

**Published:** 2016-03-02

**Authors:** Christoph Schweingruber, Paolo Soffientini, Marc-David Ruepp, Angela Bachi, Oliver Mühlemann

**Affiliations:** 1 Department of Chemistry and Biochemistry, University of Bern, Bern, Switzerland; 2 Graduate School for Cellular and Biomedical Sciences, University of Bern, Bern, Switzerland; 3 IFOM-FIRC Institute of Molecular Oncology, Milan, Italy; Korea University, REPUBLIC OF KOREA

## Abstract

Proximity-dependent *trans*-biotinylation by the *Escherichia coli* biotin ligase BirA mutant R118G (BirA*) allows stringent streptavidin affinity purification of proximal proteins. This so-called BioID method provides an alternative to the widely used co-immunoprecipitation (co-IP) to identify protein-protein interactions. Here, we used BioID, on its own and combined with co-IP, to identify proteins involved in nonsense-mediated mRNA decay (NMD), a post-transcriptional mRNA turnover pathway that targets mRNAs that fail to terminate translation properly. In particular, we expressed BirA* fused to the well characterized NMD factors UPF1, UPF2 and SMG5 and detected by liquid chromatography-coupled tandem mass spectrometry (LC-MS/MS) the streptavidin-purified biotinylated proteins. While the identified already known interactors confirmed the usefulness of BioID, we also found new potentially important interactors that have escaped previous detection by co-IP, presumably because they associate only weakly and/or very transiently with the NMD machinery. Our results suggest that SMG5 only transiently contacts the UPF1-UPF2-UPF3 complex and that it provides a physical link to the decapping complex. In addition, BioID revealed among others CRKL and EIF4A2 as putative novel transient interactors with NMD factors, but whether or not they have a function in NMD remains to be elucidated.

## Introduction

The understanding of cellular mechanisms at the molecular level requires the elucidation of protein-protein interactions *in vivo*. For large multi-factor complexes assembled on mRNAs, co-immunoprecipitation (co-IP) assays often identify many interactors that are only peripheral components and thus complicate the interpretation of such results with the risk that the plethora of apparent interactors might conceal truly insightful mechanistic connections. For example, the co-IP of UPF1, a central factor in the nonsense-mediated mRNA decay (NMD) pathway, followed by explorative mass spectrometry previously revealed a large number of interactors [1] of which only few could supposedly be directly involved in the NMD pathway or be direct interactors. Furthermore, many RNA-binding proteins can dissociate from their bound RNAs during cell lysis and reassemble haphazardly on RNA in lysates [2, 3]. Hence, RNA-dependent interactors might not always reflect truly relevant interactions *in vivo*. Besides such post-lysis rearrangements confounding the picture, functionally important interactions that occur only transiently or with low affinity might be missed in co-IP assays depending on the specific conditions used for cell lysis, affinity binding and washing. Given these limitations of conventional co-IPs, we sought for an alternative and complementing method to identify additional proteins interacting with known NMD factors.

In vertebrates, at least nine protein factors (UPF1, UPF2, UPF3B, SMG1, SMG5, SMG6 SMG7, SMG8, SMG9) were convincingly shown to be involved in the degradation of nonsense mRNA and some additional proteins have been only implicated more recently in NMD, including GNL2, SEC13, DHX34, NBAS, MOV10 [4–8]. The interactions and connectivity among these components is partly elucidated by mutational studies coupled with co-IP or yeast-two-hybrid assays. Although some NMD factors bind mRNA probably already in the nucleus [3, 9–11], the decision to elicit NMD is translation-dependent and mechanistically linked to translation termination [12, 13]. Consistent with this view, a sub-complex consisting of SMG1, UPF1, and the eukaryotic release factors ERF1 and ERF3A could be co-immunoprecipitated (called SURF)[14]. Further, the SMG1 PIKK kinase phosphorylates and activates the UPF1 helicase, and both activities are required for destabilization of the mRNA. This is thought to occur after UPF2 joins the complex, resulting in a rearrangement of the complex and favoring an active SMG1 kinase conformation [15]. UPF1 phosphorylation sites supposedly act as accessory sites for proteins that serve as adapters for general decay factors (e.g. SMG5-SMG7 recruiting the CCR4-NOT complex, which has deadenylase activity; [16]) or in the case of the endonuclease SMG6 cleave the RNA directly [17, 18]. How the other factors are integrated into the mechanism is unknown so far.

**Fig 1:**
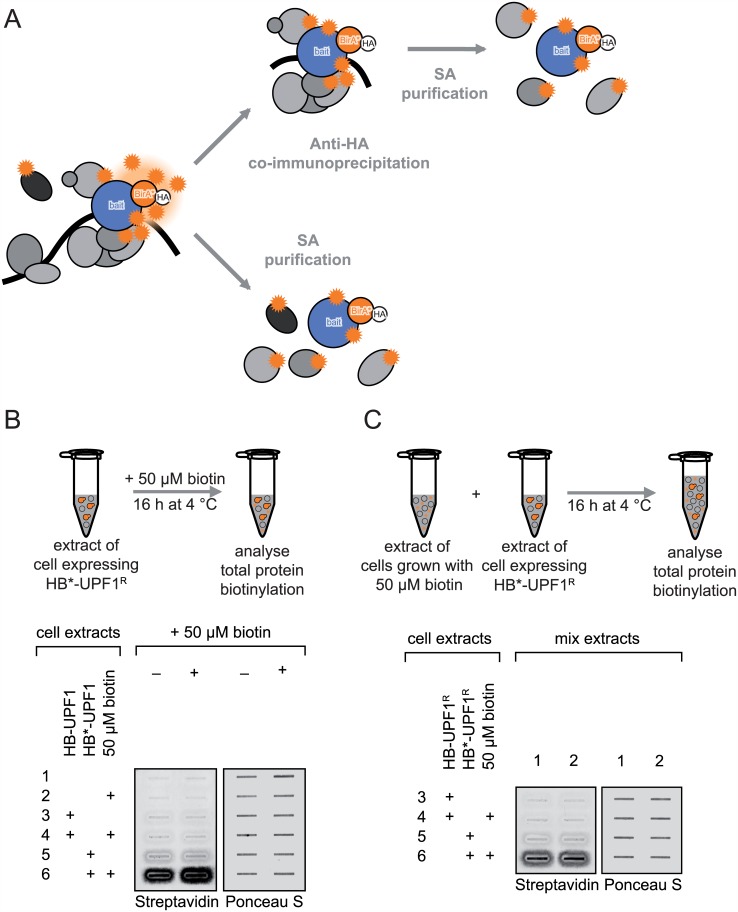
BioID assay set-up. **(A)** Expression of the promiscuous biotin ligase BirA(R118G) (HB*, orange circle) fused to a protein of interest (designated “bait”, blue circle) results in distant-dependent biotinylation (represented as orange stars) of proteins located in the vicinity of the HB*-bait protein if biotin is supplement to the cell culture. Biotinylated proteins are then affinity captured by magnetic streptavidin beads. Since the HB* fusion constructs contain a hemagglutinin (HA) tag (white circle), the BioID assay can be combined with anti-HA immunoprecipitation followed by streptavidin affintiy purification (SA) to specifically capture biotinylated proteins in the immunoprecipitate. **(B, C)** Biotinylation by HB* occurs mainly *in vivo* but not in the lysate, because neither supplementing biotin in extracts containing HB*-UPF1 (B) nor addition of an extract primed with biotin (C) boosts total protein biotinylation. 293T cells expressing either no (1, 2), wild-type BirA (HB)-tagged (3, 4) or HB*-tagged UPF1 (5, 6) were incubated without (1, 3, 5) or with 50 μM biotin (2, 4, 6) for 16 hours before extracts were prepared by gentle hypotonic lysis. In contrast to HB*, HB only biotinylates the specific biotin acceptor peptide and is used here as a negative control. (B) Where indicated, extracts were then supplemented with 50 μM biotin and incubated for 16 hours at 4°C before SA purification. (C) Extracts 1 and 2 were mixed with the HB-UPF1 (3, 4) and HB*-UPF1 (5, 6) expressing extracts followed by incubation for 16 hours at 4°C. The total biotinylation was monitored by probing with Streptavidin 800CW after slot blotting of samples onto nitrocellulose membranes.

As an alternative to co-IPs, we addressed here the protein-protein interaction network for key factors in the NMD pathway in a distant-dependent manner by a combination of IP and BioID [19, 20]. In BioID, the mutant *E*. *coli* biotin-protein ligase BirA_R118G_ (hereafter called BirA*) is fused to the bait protein and biotinylates proximal proteins promiscuously. In contrast to the wild-type BirA, which coordinates the reactive intermediate 5’-biotinoyl-AMP until binding of the specific biotin adaptor peptide onto which the biotinoyl group is transferred, the BirA* mutant releases the reactive 5’-biotinoyl-AMP and the biotinoyl group is transferred unspecifically to available primary amines in its surrounding [21]. Hence interactors residing close to the bait *in vivo* can be enriched by streptavidin (SA) purification and identified by mass spectrometry. In addition to direct BioID using known NMD factors as HA-BirA*-tagged bait proteins, we also attempted in this study a tandem purification of NMD-related factors by combining the BioID approach with co-IP to specifically identify proteins that reside closely to the bait and interact stably with it (Fig 1A). The results of these two approaches revealed new putative NMD-associated proteins that presumably interact only transiently with the NMD complex, such as the signaling factor CRKL and translation initiation factor EIF4A2. Furthermore, we corroborated the stable interactions among the NMD factors UPF1-3. We also found that SMG5 interacts with the decapping complex through protein-protein contacts, suggesting a molecular link between SMG5 and the decapping complex.

## Results

### *Trans*-biotinylation occurs exclusively *in vivo*

Promiscuous biotinylation by BirA*-tagged bait proteins to identify proteins that are *in vivo* located in the proximity of the bait (BioID) has not yet been used broadly and a careful setup of the method, including tests for sensitivity and specificity, was therefore crucial to minimize the risk for technical artefacts. First we tested if BirA*-mediated promiscuous biotinylation can also occur post lysis in our assay. Post-lysis rearrangement is a common and often neglected problem in co-IPs: proteins dynamically dissociate and reassemble into complexes in the cell lysate, thereby confounding the original compositions of these complexes in intact cells [2]. In contrast, the enzymatic *trans*-biotinylation by BirA* permanently marks proteins before lysis in intact cells and because it depends on two substrates (biotin and ATP) that are only available in small amounts in the lysate, biotinylation after lysis should not occur in BioID. Consistent with this expectation, we could not detect any biotinylation occuring in the lysate under our assay conditions, neither when biotin was directly added to the lysate (Fig 1B) nor when it was supplemented from other cell lysates (Fig 1C). As a negative control, we included the wild-type BirA fusion proteins that can only biotinylate its specific acceptor peptide. Accordingly, these lysates do not accumulate biotinylated proteins in either assay (Fig 1B and 1C). This confirms that biotinylation occurs exclusively *in vivo* and that therefore BioID identifies only factors that are located proximal to the BirA*-bait fusion protein in intact cells.

### The HA-BirA fusion proteins are functional in NMD

**Fig 2:**
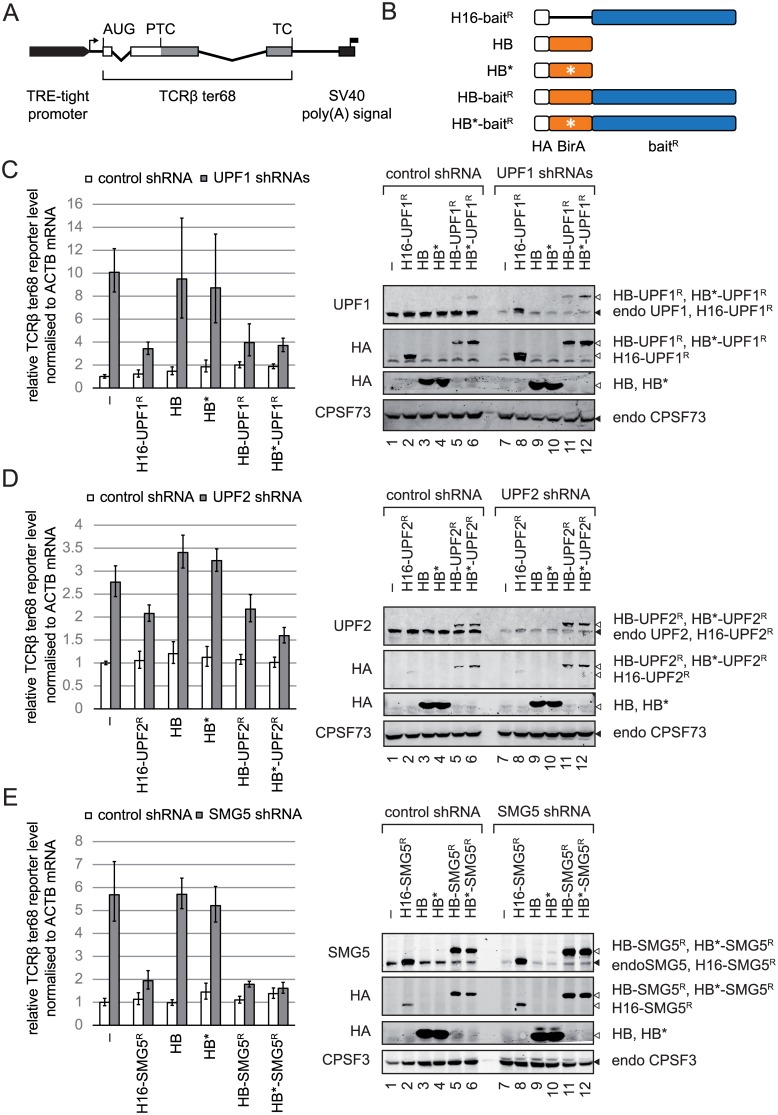
The HB*-tagged UPF1, UPF2, and SMG5 constructs are functionally active in the NMD pathway. **(A)** Schematic of the TCRβ ter68 NMD reporter construct stably integrated in HeLa Tet-Off cells [24]. The TRE-tight promoter (thick black arrow), the transcription start site (thin arrow) and the SV40 polyadenylation site (black box with flag) are indicated. AUG denotes the beginning of the open reading frame (box), and its sites of premature and normal termination are marked by the premature termination codon (PTC) and the termination codon (TC), respectively. The two introns are depicted as kinked lines **(B)** Schematic representation of the tested protein constructs. The HA-tag followed by a Gly_16_ spacer is shown as white box, BirA as orange box (with the R118G mutation denoted as white star) and bait factors as blue boxes **(C-E)** Knockdown of the indicated NMD factors was achieved by transiently transfecting the HeLa Tet-Off TCRβ ter68 cells with shRNA expressing plasmids and rescue of NMD activity was attempted by co-transfection of the expression plasmids encoding the indicated RNAi resistant (denoted ^R^) constructs. Relative TCRβ ter68 RNA levels, normalized to ACTB mRNA, were measured by real-time qRT-PCR (left side) and quantified by the ΔΔCT method. The columns represent the average expression level of three independent biological replicates. Error bars mark the cumulated variation among the ΔΔCT values in the biological replicates. The levels of the indicated proteins were analyzed by western blotting (right side). (C) Knockdown of endogenous UPF1 can be rescued by expressing H16-UPF1^R^, HB-UPF1^R^ or HB*-UPF1^R^ fusion proteins, (D) knockdown of endogenous UPF2 can be rescued by expressing H16-UPF2^R^, HB-UPF2^R^ or HB*-UPF2^R^ fusion proteins, and (E) knockdown of endogenous SMG5 can be rescued by expressing H16-SMG5^R^, HB-SMG5^R^, and HB*-SMG5^R^ fusion proteins. Positions of recombinant and endogenous proteins on the blots are indicated on the left by white and black triangles, respectively.

BioID has been applied in the context of rather stable cellular structures, such as the nuclear lamina [19], the centrosome [22] or the nuclear pore complex [23], but has so far not been used to explore highly dynamic interaction networks among mRNP components, such as for example the NMD factors. It was therefore important to verify that the presence of BirA* did not disturb the fused NMD factors in their functionality in the NMD pathway before attempting SA purifications. To this end, we monitored whether the BirA-fusion proteins could functionally rescue the depletion of their endogenous counterparts. As readout for NMD activity, we measured the mRNA level of the stably integrated TCRβ ter68 NMD reporter gene [24](Fig 2A) by reverse transcription followed by quantitative polymerase chain reaction (RT-qPCR). The endogenous NMD factor was depleted by RNA interference and an RNAi-resistant version of the corresponding BirA*-tagged NMD factor or corresponding control constructs were expressed (Fig 2B). Upon shRNA-mediated depletion of UPF1, the TCRβ ter68 mRNA steady-state levels increased by about ten fold compared to a control knockdown. The overexpression of shRNA-resistant (denoted by ^R^) HA-Gly_16_ (H16-), HA-BirA (HB-), or HA-BirA(R118G) (HB*-) tagged UPF1 at near endogenous levels restored NMD, as indicated by the reduction of the TCRβ ter68 reporter mRNA levels, while neither the expression of free HB nor HB* could restore NMD (Fig 2C). In principle, one could imagine that extensive surface biotinylation of the HB*-UPF1 fusion protein might impair its molecular function. However, we find that both HB-UPF1 and HB*-UPF1 fusion protein can restore NMD comparably, indicating a sufficient pool of functional HB*-UPF1 under our assay conditions. Similarly, we find that the presence of the promiscuous HB* and HB*-UPF1 in the cell and potential background biotinylation did not prevent NMD.

The identical setup was used to test the ability of the BirA*-UPF2^R^ constructs (Fig 2D) and BirA*-SMG5^R^ constructs (Fig 2E) to restore NMD. Similarly to UPF1, depletion of UPF2 or SMG5 increased the TCRβ ter68 reporter mRNA and expression of the RNAi-resistant UPF2^R^ or SMG5^R^ construct again lowered the reporter RNA level, indicating that these two NMD factors were also functional when tagged with BirA*.

In summary, we could show that the H16-, HB-, and HB*-tagged bait constructs of UPF1, UPF2, and SMG5 are still functional in NMD. We further gained evidence that surface biotinylation of the bait construct does not prevent the restoration of NMD and that the potential bulk biotinylation by free HB* does not detectably interfere with NMD.

### BioID detects known interactors as well as novel ones

**Fig 3:**
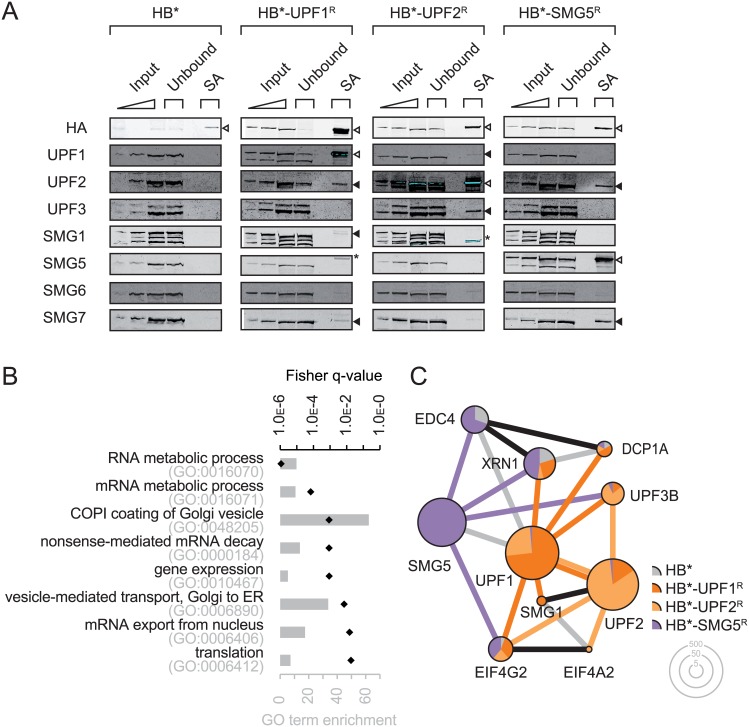
BioID using HB*-UPF1^R^, HB*-UPF2^R^ and HB*-SMG5^R^ captures known and novel interactors. **(A)** HB*, HB*-UPF1^R^, HB*-UPF2^R^, and HB*-SMG5^R^ were transiently expressed in 293T cells at similar levels as the endogenous factor. On the western blots, the HB*-UPF1^R^, HB*-UPF2^R^ and HB*-SMG5^R^ fusion proteins can be distinguished from their respective endogenous proteins by their slower migration. The biotinylated proteins were isolated by streptavidin affinity capture and analyzed by western blotting for interaction among the NMD factors indicated on the left. The same samples were also analyzed by mass-spectrometry. Putative interactors were identified by excluding proteins that were not detected by more than two times the total normalized spectra counts in isolates of at least one HB*-bait factor when compared to the control isolates from cells expressing free HB*. **(B)** Overrepresented biological processes in the combined dataset were identified by Gene ontology (GO) analysis performed with enrichnet.org [78]. Only significantly enriched parental GO terms are shown (Fisher q-value ≤ 0.05). Grey bars represent GO term enrichment (frequency in query / frequency in human genome), black points represent the corrected Fisher q-value. **(C)** The identified putative interactors were analyzed by the PPI spider tool [34] for the enrichment of a protein-protein subnetwork using the experimental data in the IntAct database. This analysis revealed the significantly enriched (p < 0.01) network of binary interactors. The factors are depicted as pie chart. The diameter of the pie charts is proportional to the logarithmic total normalized spectrum count obtained from the LC-MS/MS analysis. The segments correspond to the fraction of the total normalized spectrum counts from the individual BioID samples for this factor. The connecting lines between the factors are colour-coded according to samples in which they were detected. Black lines denote direct interactions and grey lines connect human factors whose interaction is documented in the IntAct database.

Next we wanted to identify proteins that are accessible to proximity dependent *trans*-biotinylation by HB*-UPF1^R^, HB*-UPF2^R^, and HB*-SMG5^R^. We expressed the HB*-bait fusion constructs at near endogenous levels and allowed biotinylation for 16 hours in the presence of 50 μM biotin. The biotinylated proteins were purified over magnetic streptavidin beads, resolved on polyacrylamide gels and either processed for mass spectrometric analysis (see below) or used for western blotting using antibodies of known interactors of the tested NMD factors (Fig 3A). The proteins in the SA-purified fractions indeed contained many of the known close interactors, but not more distant complex components. For example, HB*-UPF1^R^ selectively biotinylated UPF2, but not UPF3B, which appears to be more distant in the complex based on the cryo-EM structure models published by Melero and colleagues [25]. Biotinylated SMG1, SMG6 and SMG7 were observed just above the detection limit, whereas SMG5 was undetectable by western blotting in the HB*-UPF1^R^ sample. We could confirm additional previously documented direct interactions, namely HB*-UPF2^R^ with biotinylated UPF3B [26, 27] and HB*-SMG5^R^ with biotinylated SMG7 [16, 28, 29].

**Fig 4:**
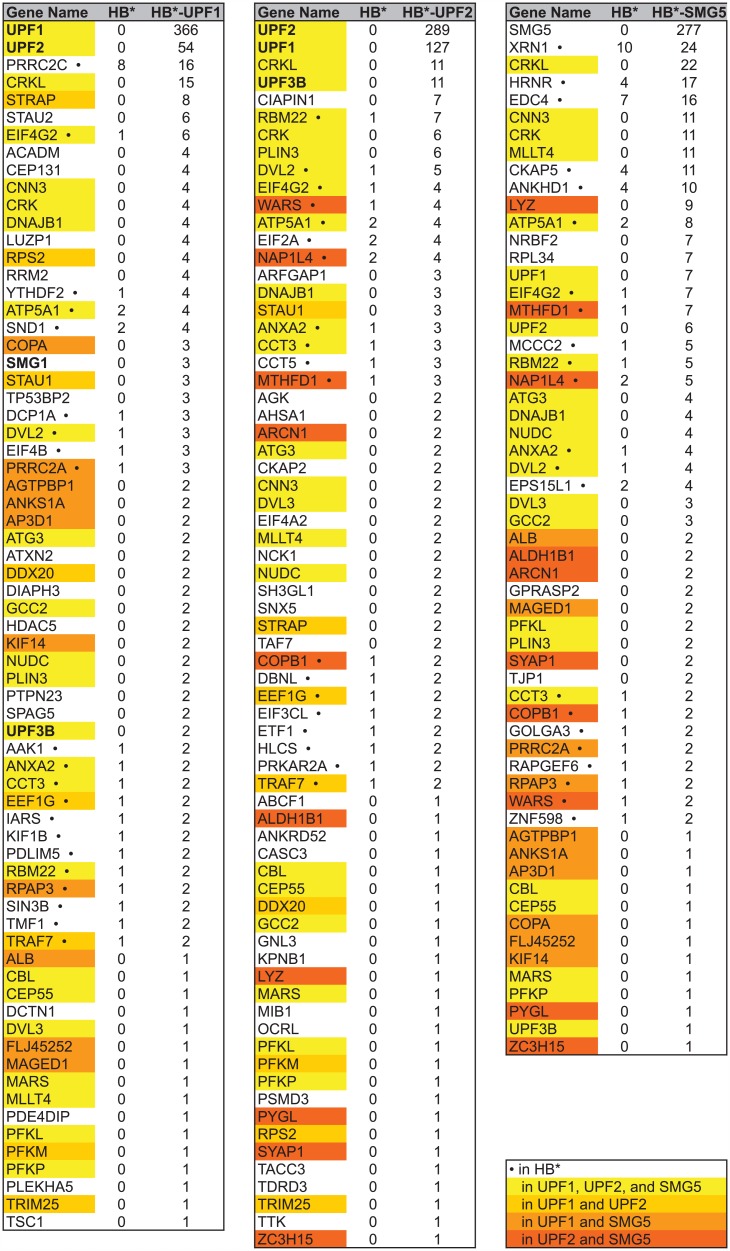
Lists of proteins identified by BioID using HB*-tagged UPF1, UPF2 and SMG5 as a bait. The normalized spectral counts obtained with the indicated HB*-tagged bait or the HB* alone (specificity control) are shown for each of the identified proteins. Proteins also detected in the HB* control are marked by a black dot, proteins identified by more than one NMD factor are highlighted by the indicated color code.

As mentioned above, the SA-purified proteins were also subjected to mass spectrometric analysis using liquid chromatography tandem mass spectrometry analysis (LC-MS/MS). Proteins were excluded from our analysis, i) if they were identified with less than two exclusive unique peptides, ii) if they were common contaminants, or iii) if the normalized spectrum count in the HB* control sample exceeded half the normalized spectrum count in all the HB* fusion protein samples. The remaining proteins that we identified by LC-MS/MS are listed in Fig 4. Of the totally 97 proteins identified in the three SA pulldowns, 15 factors were biotinylated by all three baits (marked in bright yellow) and another 15 by two of the three baits (different shades of orange). Some of them were only detected with few spectral counts, indicating that they either might be low abundant, or that they are associated with the NMD complex only very transiently or peripherally. In contrast, the two related signaling factors CRKL and CRK and the heat shock protein DNAJB1 were biotinylated quite efficiently and specifically by HB*-UPF1^R^, HB*-UPF2^R^, and HB*-SMG5^R^.

Gene ontology (GO) analysis on biological processes on our combined dataset confirmed a significant enrichment in nonsense-mediated mRNA decay (Fisher *q*-value = 8.7e-04) and coupled to this an enrichment in RNA and mRNA metabolic processes (*q* = 1.1e-06 and *q* = 6.8e-05, respectively), in mRNA export (*q* = 1.5e-02) and in translation (*q* = 1.8e-02; Fig 3B). Consistently, on a higher hierarchy level of the GO terms, post-transcriptional regulation of gene expression was highly enriched (*q* = 8.8e-04; not shown). In addition, we also found genes of the GO categories COPI-coating of Golgi vesicles and retrograde vesicle-mediated transport from Golgi to endoplasmatic reticulum (ER) to be enriched (*q* = 8.7e-04 and *q* = 7.0e-03, respectively), suggesting a physical proximity between Golgi vesicles and the tested NMD factors. This finding would fit with the emerging evidence suggesting a mechanistic link between mRNA and membrane trafficking (reviewed in [30]). Moreover, the recently identified NMD factors NBAS and SEC13 [4, 5, 31] have previously been reported to associate with retrograde transport complexes from Golgi to ER [32, 33]. However, we did not detect NBAS or SEC13 in our analysis.

To understand how the purified biotinylated proteins integrate into the existing protein-protein interaction (PPI) networks, we utilized the PPI spider tool [34] and found that we significantly enriched (*p* < 0.005) a sub-network of known binary protein-protein interactions from the IntAct database consisting of NMD factors, of components of the 5’-3’ exonucleolytic RNA degradation pathway, and of translation initiation factors (Fig 3C). The graphical summary of this analysis shows that the tested NMD factors (UPF1, UPF2 and SMG5) biotinylated factors of these three sub-networks differentially: SMG5 shows a strong connection to the 5’-3’ exonucleolytic pathway and to translation by purification of XRN1, EDC4 and DCP1, and EIF4G2, respectively. On the other hand, its association with the other NMD factors (UPF1, UPF2, UPF3B and SMG1) seems to be rather weak. *Vice versa*, UPF2 confirmed tight links to with UPF1 and UPF3B, as well as a connection with the translation initiator EIF4G2, but comparably weak links to the 5’-3’ exonucleolytic pathway (XRN1 and DCP1A). Finally, UPF1 stands somewhere in between SMG5 and UPF2, exhibiting strong links to the NMD factors UPF2 and SMG1 (but only a weak link to UPF3B), and associations to both translation initiation (EIF4G2) and 5’-3’ exonucleolytic RNA degradation (XRN1, DCP1A). By and large, these results are in good agreement with the known functions of UPF1, UPF2 and SMG5 in NMD and with the current models for NMD [35, 36].

### Combination of BioID with co-IP enriches interactors mediated by the mRNP

**Fig 5:**
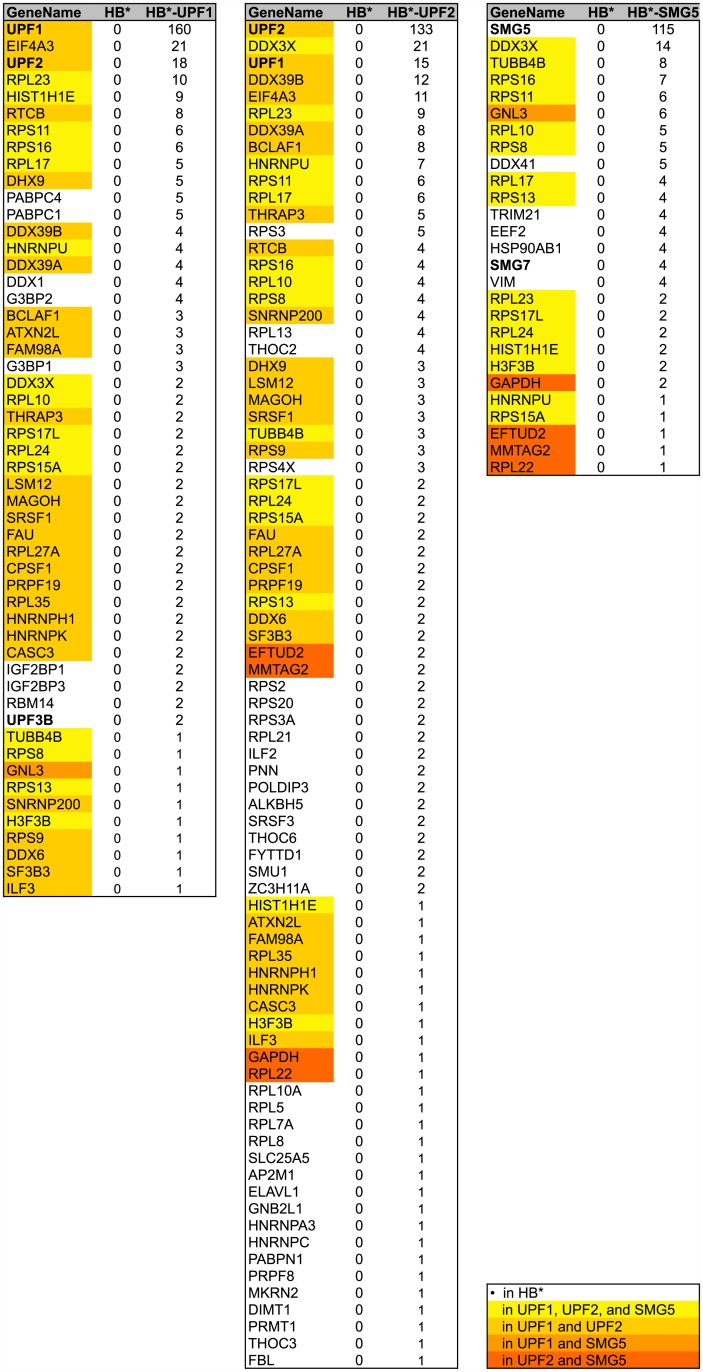
Lists of proteins identified with the combined BioID / anti-HA immunoprecipitation approach using HB*-tagged UPF1, UPF2 and SMG5 as a bait. The normalized spectral counts obtained with the indicated HB*-tagged bait or the HB* alone (specificity control) are shown for each of the identified proteins. Proteins identified by more than one NMD factor are highlighted by the indicated color code.

As outlined above, BioID functions fundamentally different from co-IPs in that it appends biotin to nearby proteins in a distance- and time-dependent manner, whereas co-IPs reveals interactors forming a physical complex with the bait that is stable enough to endure the purification conditions. Tandem purification by co-IP followed by SA purification (Fig 1A) should therefore enrich for proteins that reside proximal to our baits in a stable complex. This tandem purification approach should also exclude proteins that are biotinylated at background level. Using anti-HA magnetic beads, we precipitated biotinylated proteins that were in complex with HB*, HB*-UPF1, HB*-UPF2, and HB*-SMG5 and analyzed them by LC-MS/MS. As in our straight SA purifications described above, we excluded proteins from the analysis that were common contaminants, that were identified with less than 2 unique peptides, or if the normalized spectrum count in the HB* control sample exceeded half the normalized spectrum count in all the HB* fusion protein samples. Using to these criteria, we identified a total of 78 interactors in the three samples (Fig 5). Besides 7 ribosomal proteins of the small and large ribosome subunits, the DEAD box RNA helicase DDX3X was identified with all three bait proteins. There is evidence that DDX3X enhances translation initiation on viral and cellular mRNAs with extensive secondary structures in the 5’ UTR [37–39] and that it is a constituent of mRNPs, interacting with poly(A)-binding protein PABPC1, the export factor NXF1 and with several translation initiation factors (EIF4E, EIF4G1 and subunits of EIF3; [39–41]). Mutations in DDX3X are associated with lymphocytic leukemias, WNT signaling and and intellectual disability [42, 43].

**Fig 6:**
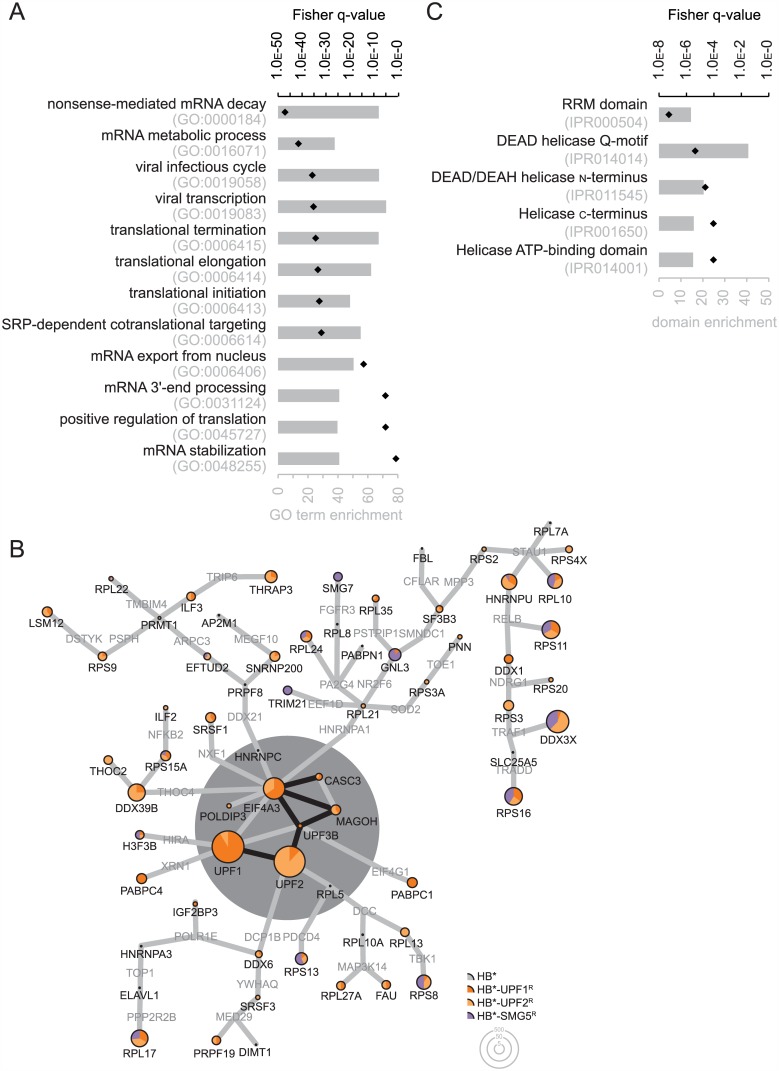
Among the interactors identified by the combined BioID / anti-HA immunoprecipitation approach using HB*-UPF1^R^, HB*-UPF2^R^ and HB*-SMG5^R^, mRNP components are enriched. HB*, HB*-UPF1^R^, HB*-UPF2^R^, and HB*-SMG5^R^ were transiently expressed in 293T cells as in Fig 3. Protein biotinylation was boosted by addition of 50 μM biotin for six hours before harvesting. From the cell extracts, bait-containing complexes were immunoprecipitated using magnetic anti-HA beads followed by SA capture of the biotinylated proteins in these precipitates. The isolated proteins were resolved by NuPAGE and identified by mass spectrometry. **(A)** Overrepresented biological processes among the 78 common interactors of HB*-UPF1^R^, HB*-UPF2^R^ and HB*-SMG5^R^ were identifed by GO analysis as in Fig 3B. **(B)** The common interactors identified in the tandem purified samples were analyzed by the PPI spider tool as in Fig 3C. They enrich significantly in a binary protein-protein interaction network (central grey zone) including the UPF1-UPF2-UPF3B (UPF) complex and the exon-exon junction complex (EJC). An extended network (outside the grey zone) connecting general mRNP components and ribosomal proteins is still significantly enriched (p < 0.01) if a single gap (nods depicted by grey gene names) is tolerated between the identified proteins (nods with pie charts). **(C)** The proteins identified in the combined BioID/co-IP are enriched in domains and motifs that are typical for RNA-binding proteins. These enriched domains of the InterPro database were identified by GO analysis using the tools on enrichnet.org [78] as described in Materials & Methods.

We find that the combined interactors of the tandem purified baits UPF1, UPF2 and SMG5 are most significantly enriched in biological processes that encompass general mRNA metabolic processes including NMD, as well as different GO-terms relating to translation (Fig 6A), consistent with NMD being mechanistically coupled to translation. Also, the combined interactors of the tandem purification integrate into existing protein-protein interaction networks with high significance (Fig 6B). Using the PPI spider tool [34], we enriched with high significance (*p*-value < 0.005) a sub-network of binary interactions that comprises UPF1, UPF2 and the exon-exon junction complex (EJC; represented by EIF4A3, CASC3 and MAGOH). This core network has the highest overlap among the hits of the direct SA purification and of the tandem purification for the three tested NMD factors. As in the straight BioID approach, SMG5 was not detected in the HB*-UPF1 and HB*-UPF2 samples in the tandem purification, suggesting that SMG5 interacts with the UPF complex only transiently and/or substoichiometrically, or that it is not accessible to biotinylation when associated with the UPF complex. Likewise, no EJC factors were biotinylated by HB*-SMG5.

The core network consisting of reported direct PPI can be extended if one gap between two identified proteins is allowed (i.e. one protein that was not detected in our analysis can link two identified proteins in the PPI network). This extended network (Fig 6B) is still statistically significant compared to fitting random proteins into the network (*p*-value < 0.05). The extended network comprises additional RNA-binding proteins known to be involved in general mRNA metabolism, mRNP components, and ribosomes. Notably, we find the nuclear export protein DDX39B/UAP56 and the THOC2 interacting with the UPF1-UPF2-EJC complex, consistent with our previously described association between UPF1 and the TREX complex [1]. Many of the interactions in the expanded network appear to be mediated by scaffolding RNA (presumably mRNA and rRNA) rather than representing direct protein-protein interactions. Evidently, peripheral interactors in the extended network are only connected to the core network through eleven ribosomal proteins (Fig 3B). Furthermore, the interactors are significantly enriched in domains and motifs common for RNA binding according to InterPro annotations (Fig 6C). In contrast, no significant enrichment for any particular domain was detected in the direct SA purification (not shown). This difference would be in line with our expectation that the interactors identified in the tandem purification (Fig 6) are mRNP components that are quite stably associated with the mRNA interaction, whereas the proteins identified in the single SA purification (Fig 3) would mainly represent factors that associate only weakly or transiently with the NMD factors.

### Interaction of the NMD factors UPF1 and SMG5 with decapping factors

In the mass-spectrometric analysis of the BioID experiment, we could dectect the 5’-3’ exonucleolytic factors DCP1A and XRN1 above background in the HB*-UPF1 and HB*-SMG5 samples. Interestingly, EDC4 was only purified with HB*-SMG5^R^ but not with HB*-UPF1^R^ or HB*-UPF2^R^, suggesting different availability to *trans*-biotinylation. UPF1 has been traditionally associated with the decapping factors both in yeast and animal cells [44, 45] and although all NMD factors co-localize microscopically with decapping factors in p-bodies [46], only UPF1 and SMG5 have been shown to co-purify with DCP1A and PNRC2 (a probable orthologue of yeast Edc1p and Edc2p) in human cells [44, 46, 47]. Hence we aimed to further characterize the interaction of the NMD complex with the decapping complex by co-immunoprecipitation of overexpressed H16-UPF1^R^ and H16-SMG5^R^ with magnetic anti-HA beads.

**Fig 7:**
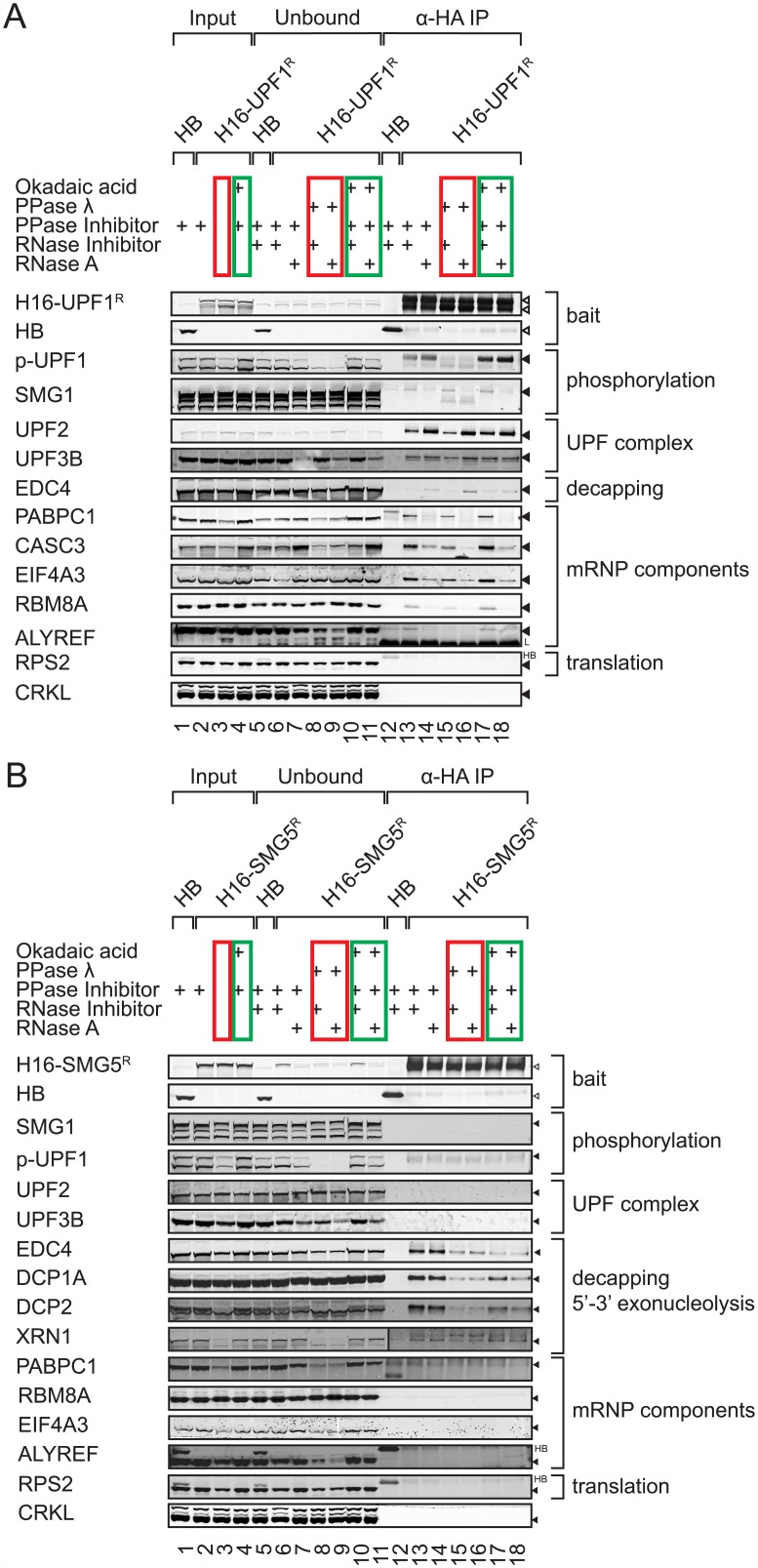
Interaction of the NMD factors UPF1 and SMG5 with mRNP components and decapping factors. Western blots were performed to detect proteins that co-immunoprecipitate with H16-UPF1^R^ **(A)** or H16-SMG5^R^ **(B)** overexpressed in 293T cells. To boost the phsphorylation state of proteins, cells were treated with okadaic acid for 3 hours before harvesting and phosphatase (PPase) inhibitors were added to the lysates (lanes 4, 10, 11, 17, 18; highlighted by green retangles). To dephosphorylate proteins, cell lysates were incubated with PPase λ prior to IP (lanes 3, 8, 9, 15, 16; red rectangles). Finally, the RNA dependence of the associated proteins was assessed by RNase A treatment of lysates (lanes 7, 9, 11, 14, 16, 18). Input, unbound (5x10^5^ cell equivalents each), and immunoprecipitated material (equivalent to 1x10^7^ cells) were separated on SDS-PAGE, transferred to nitrocellulose membranes and probed with antibodies against the indicated proteins. Exact postions of overexpressed and endogenous proteins are indicated by white and black triangles, respectively, L denotes signal originating from the antibody light chain and HB bleed-through signal from the HA-BirA protein. For better visibility, a higher intensity scan of the membrane piece is shown in (B) for the XRN1 IP samples.

In the co-immunoprecipitates of H16-UPF1^R^, we found UPF2 and UPF3B irrespective of protein phosphorylation or treatment with RNase A (Fig 7A) in accordance with previous studies. UPF1 also interacted with other general mRNP components including PABPC1 and the EJC factors CASC3 (MLN51), EIF4A3 and RBM8A (Y14). The interaction of UPF1 with PABPC1 was fully and with the EJC partially mediated by RNA, and dephosphorylation of the lysate with protein phosphatase λ (PPase λ) slightly reduced the interaction of the EJC factors with UPF1. Furthermore, less SMG1 co-precipitated with UPF1 when the lysate was treated with RNase A, indicating that this interaction might be at least partially mediated by RNA (Fig 7A).

In the combined BioID/co-IP approach, several ribosomal proteins of the small subunit were detected (Fig 5) and in yeast UPF1 has been shown to interact with RPS26 [48]. However, the 40S ribosomal subunit did not co-IP in detectable amounts with H16-UPF1^R^ as exemplified by the absence of RPS2, implying that if UPF1 indeed associates with the 40S ribosomal subunit in human cells, this interaction is presumably weak and/or transient.

Likewise, the signaling factor CRKL, which was biotinylated by HB*-UPF1, HB*-UPF2 and HB*-SMG5 (Fig 4), could not be detected in the UPF1 IP (Fig 7A) nor in the SMG5 IP (Fig 7B), indicating a weak and/or transient association with UPF1 and SMG5.

In contrast to H16-UPF1^R^, H16-SMG5^R^ robustly and reproducibly co-purified decapping factors EDC4, DCP1A, DCP2, and XRN1 in an RNase A resistant manner (Fig 7B). Interestingly, treatment of the lysate with PPase λ reduced the amount of co-purifying decapping factors, suggesting that protein phosphorylation stabilizes the association of the decapping complex with SMG5. Corroborating earlier indications that the association of SMG5 with the mRNP and with UPF1 is probably labile or limited by SMG7 levels [16, 49], we failed to co-purify detectable levels of common mRNP components (PABPC1, RBM8A, EIF4A3, ALY/REF), NMD factors (UPF1, UPF2, UPF3B, SMG1) or RPS2 with overexpressed H16-SMG5^R^.

### UPF2 transiently interacts with components of the translation machinery

**Fig 8:**
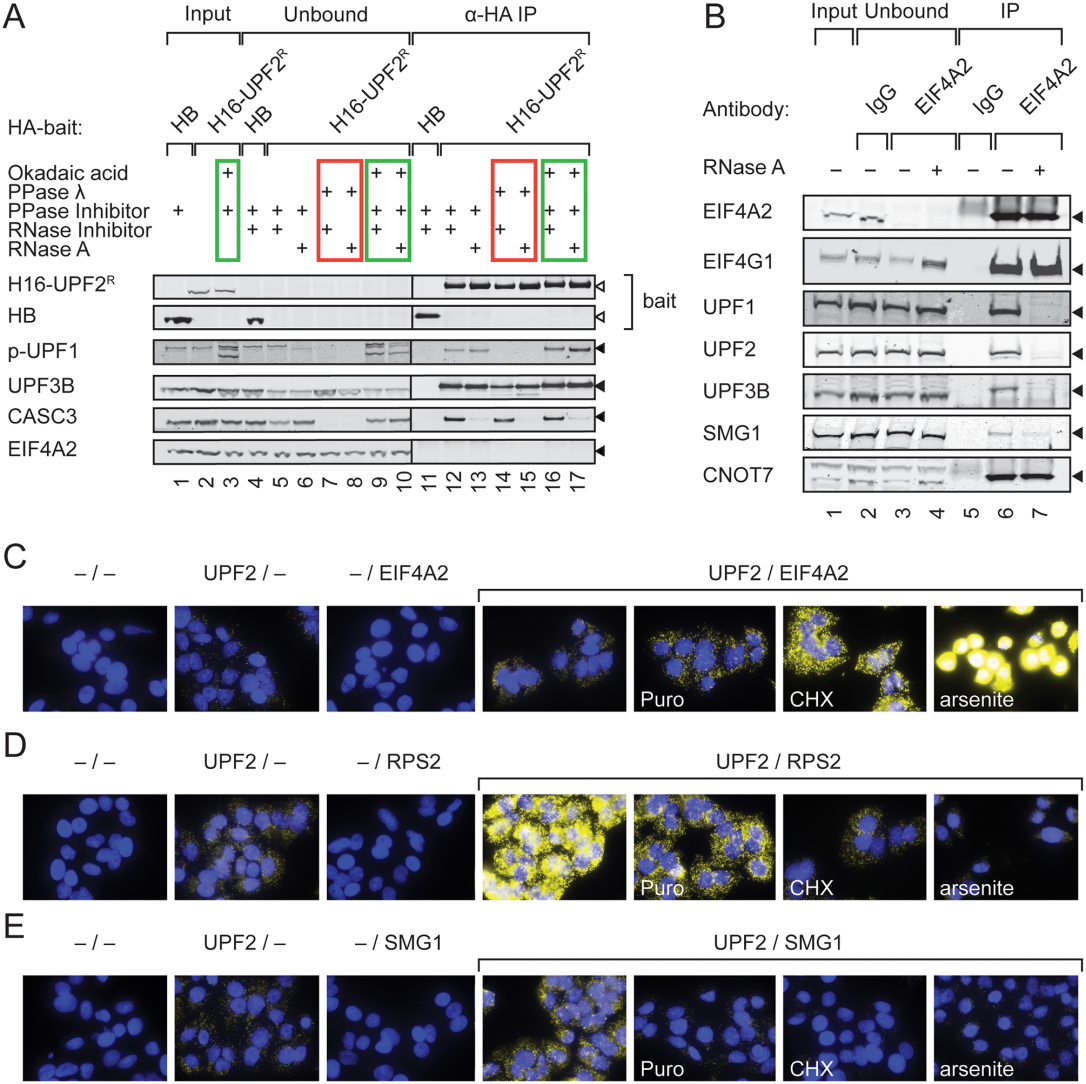
UPF2 transiently interacts with translation factors. **(A)** Western blots of anti-HA co-immunoprecipitation experiment from 293T cells overexpressing H16-UPF2^R^. Protein hyperphosphorylation, dephosphorylation and RNase A treatment conditions were as in Fig 7. **(B)** Western blot of co-immunoprecipitation of endogenous EIF4A2 from 293T cells with an antibody against the n-terminus of EIF4A2 (lanes 3,4, 6,7) either with (lanes 4 and 7) or without RNase A treatment (lanes 3 and 6). Whole IgG was used as a control (lanes 2 and 5). Input, unbound (5x10^5^ cell equivalents each) and immunoprecipitated material (equivalent to 1x10^7^ cells) was used in (A) and (B). White and black triangles denote the position of the overexpressed and endogenous proteins, respectively. **(C-E)** Proximity ligation assays (PLA) probing for the effect translation inhibition on the pairwise co-localization of UPF2 and EIF4A2 (C), UPF2 and ribosomal protein RPS2 (D), and UPF2 and SMG1 (E). HeLa Tet-Off TCRβ ter68 cells (clone 2.2) were grown and where indicated treated with puromycin (Puro), cycloheximide (CHX) or arsentite. The antibody pairs for detection are depicted above the images. Omission of one or both primary antibodies controlled for the specificity of the PLA signal.

The interaction between UPF2 and the translation initiation factor EIF4A2 was detected in both purification strategies described above (although only with a single unique peptide in the tandem purification). Since EIF4A2 has been shown to co-purify with the CCR4-NOT1 deadenylation complex and to be involved in the translational repression mediated by the miRISC complex in some studies [50–52], we wanted to investigate the UPF2-EIF4A2 interaction further. In IPs with H16-UPF2^R^ as the bait, both UPF1 (detected with a phospho-UPF1 specific antibody) and UPF3B were co-purified, indicating formation of an intact UPF complex (Fig 8A). This interaction was by and large independent of RNA and the phosphorylation status of UPF1. We also co-purified in an RNase A sensitive manner the EJC component CASC3. Despite these confirmatory associations, we could not detect any co-purifying EIF4A2 with the H16-UPF2^R^ bait. However, the co-immunoprecipitation of endogenous EIF4A2 with an antibody against its N-terminus co-purified UPF1, UPF2, UPF3B, and SMG1 in a RNase A-sensitive manner, indicating that they are concurrent on the mRNP (Fig 8B). In contrast, the interaction of EIF4A2 with EIF4G1 and with the CCR4-NOT complex component CNOT7 (CAF1) was resistant to RNase A, indicating a protein-protein mediated link of EIF4A2 to both the translation initiation complex and the CCR4-NOT complex.

We reasoned that the failure to detect an interaction between the UPF2 bait and EIF4A2 in our co-IPs might be due to their association on mRNPs being only transient. To test this, we used another proximity-dependent method that should allow the detection of even transient interactions, a proximity-ligation assay (PLA) that generates a fluorescence signal if two target proteins co-localize. Indeed, we found a co-localization of UPF2 and EIF4A2 in the cytoplasm of human cells (Fig 8C). Because EIF4A2 is a translation initiation factor, its co-localization with UPF2 might depend on the translational status of the scaffolding mRNP. Therefore, we inhibited translation at different steps by specific drugs: i) arsenite causes oxidative stress and should results in the sequestration to stress granules of mRNPs that do not contain elongating ribosomes [53], ii) cycloheximide (CHX) immobilizes translating ribosomes on the mRNA during the elongation phase, and iii) puromycin (Puro) terminates and releases the nascent peptide chain [54]. Compared to untreated cells, arsenite and CHX both increased the PLA signal, indicative for an enhanced co-localization of UPF2 and EIF4A2. In contrast, puromycin, which does not prevent inital steps of translation but instead terminates it prematurely, did not led to a substantial increase in the co-localization signal. Our results therefore suggest that colocalization takes place on mRNPs before or at the stage of translation inititation and is relieved during translation elongation.

Accordingly, we also find that UPF2 colocalizes with the ribosomal protein RPS2 of the small ribosomal subunit and that inhibition of translation by either drug reduces the colocalization signal (Fig 8D). The fact that puromycin decreases the UPF2-RPS2 co-localization only partially, whereas CHX and arsenite almost completely abrogate it, indicates that UPF2 and RPS2 mainly interact during translation initiation and to some extent during the elongation phase of translation.

Finally, we also assessed which effect the three translation inhibiting drugs exert on the interaction between UPF2 and SMG1. In untreated cells, a punctate cytoplasmic co-localization signal was detected, which was lost when translation was inhibited with puromycin, CHX or arsenite (Fig 8E). This transient translation-dependent co-localisation is in agreement with current models for NMD, in which UPF2 and SMG1 come together on substrate mRNPs only after translation termination has taken place [14, 55].

Collectively, the data of the UPF2 PLA assays suggest that UPF2 associates with the mRNP before or at the latest during translation initiation and undergoes a series of transient but probably indirect interactions along the process of translation. The co-localization with the translation initiation factor EIF4A2 takes place before translation is initiated but is resolved during translation. In opposite manner, the small subunit ribosomal protein RPS2 only co-localizes upon translation initiation. The interaction with SMG1 occurs only if translation completes.

## Discussion

In search for a complementing method to co-IPs, which over the last 20 years have been abundantly used to elucidate interactions among the different NMD factors [1, 56], we used the proximity-dependent method BioID and tested it for three well characterized NMD factors UPF1, UPF2 and SMG5. *In vivo* biotinylation by the promiscuous biotin ligase BirA* fused to UPF1, UPF2 or SMG5 has the potential to identify even weak and transient interactions of these factors that would not be detected by co-IPs. We tested and compared a straight BioID protocol with a combined approach, in which only biotinylated proteins that co-immunoprecipitated with the BirA* fusion proteins were detected (Figs 1A and 3–6).

### Technical considerations for BioID

Unlike co-IPs, during which rearrangements of RNA binding proteins after lysis frequently occur and confound the results [2], we demonstrate here that BioID exclusively labels neighbouring proteins in intact cells without any biotinylation occuring after cell lysis (Fig 1B). Furthermore, it was crucial to confirm that the respective BirA* fusion protein was still functional in NMD (Fig 2), implying that it assembles into the same or at least very similar complexes as the endogenous NMD factor. The expression level of the BirA* fusion protein relative to the respective endogenous protein as well as the concentration and duration of added biotin are crucial parameters that affect the ratio between specific and background biotinylation in BioID and they need to be optimized when setting up a BioID assay. For our application, we empirically determined 50 μM biotin for 16 hours to be optimal, and we used transfection conditions that resulted in BirA* fusion protein levels that were similar to or at most 2-fold higher than the endogenous levels of UPF1, UPF2 or SMG5, respectively (Fig 3A).

### Stable structural complexes vs. dynamic associations

BioID has previously been successfully applied to characterize stable structural assemblies, such as the nuclear envelope and pore complex [19, 23] or centrosomes [22]. Here, we applied BioID to proteins involved in NMD that arguably form more transient and dynamic complexes with altering composition on the mRNP, many of which would not be expected to be captured by co-IP. Accordingly, in the BioID approach (Figs 3 and 4) we found many novel potential interactors whose association with the NMD factors was not anymore or to a lesser extent seen in the more stringent combined BioID/co-IP approach (Figs 6 and 5). An example for such a putative transient involvement in NMD is CRKL, a phospho-tyrosine and SH2-SH3 adaptor protein with a role in lymphoid signaling pathways [57, 58]). Noteworthy, CRKL was recently identified among novel RNA-binding proteins that lack any known RNA-binding domains [59]. Whether CRKL or its closely related homolog CRK have a role in NMD remains to be explored in the future.

Instead, we further investigated the apparent association of UPF2 and EIF4A2 in the mRNP, which was indicated by the straight BioID (Fig 4), and we found that the UPF complex and SMG1 co-purified in an RNase A sensitive manner in the immunoprecipitates of EIF4A2 (Fig 8B). While the EJC component EIF4A3 is a known co-activator of NMD, its paralogues EIF4A1 and EIF4A2 are components of the cap-binding EIF4F complex involved in translation initiation. Neither EIF4A1 nor EIF4A2 knockdown was found to stabilize NMD reporter mRNAs [60] [61], arguing against EIF4A2 being a *bona fide* NMD factor. However, EIF4A2 appears to mediate translational repression by miRISC [50–52], and another component of the EIF4F complex, EIF4G, was found to antagonize NMD when tethered close to a premature termination codon [62, 63], suggesting that EIF4A2 could potentially impact on NMD through one of these mechanisms. Interestingly, the association of UPF2 and EIF4A2 seems to be dynamic and can be enhanced by translation inhibitors in PLA assays (Fig 8C). In contrast, the association of UPF2 and SMG1 appears to depend on translation termination, since either translation inhibition by arsenite, cycloheximide, or puromycin treatment prevented their co-localization in the PLA assay (Fig 8C). Similarly, the co-localization with RPS2 of the small ribosomal subunit was reduced upon inhibition of translation and abolished under arsenite treatment. Overall, these data suggest a dynamic association or re-distribution of either factor on mRNP during translation.

### Association of UPF1 and SMG5 with decapping factors

SMG5 was previously shown to associate with DCP1A and PNRC2 [47], unlike SMG6 and SMG7, and the NMD factors were reported to co-localize with decapping factors in p-bodies [64]. Our BioID results indicate that UPF1 and SMG5 are physically close to the decapping factor DCP1A and the major cytoplasmic 5’-3’ exonuclease XRN1, respectively (Figs 3C and 4). Moreover, HB*-SMG5 also biotinylated EDC4 which forms a molecular scaffold for the decapping complex and XRN1. Further, we co-purified decapping factors DCP1A, DCP2, and EDC4, as well as XRN1 with H16-tagged SMG5 in conventional IPs in a manner insensitive to RNase A treatment, corroborating a direct molecular link among UPF1, SMG5, and the decapping complex in human cells. The association between SMG5 and DCP1A, DCP2, EDC4 and XRN1 seems to be enhanced by protein phosphorylation, because dephosphorylation of the lysate reduced the association (Fig 7B). However, also treatment of cells with okadaic acid before the IP prevented a fraction of decapping factors from associating with SMG5, indicating that there might exist phosphorylation states in one or several of these factors that precede their association with SMG5 and that negatively impact on the SMG5-decapping complex interaction. We did not further determine the responsible phosphorylation sites, but it is noteworthy that similar inhibitory phosphorylation sites have been described for the decapping factors [65, 66]. In addition, the phosphorylation status of UPF1 was inferred to determine the association with decay factors [47, 67] and hence might modulate the association with 5’-3’ exonucleolytic decay factors.

In light of the previously published functional data, it appears that SMG5-mediated mRNA degradation depends on activation by its cofactor SMG7: degradation of a reporter mRNA by tethering of SMG5 to it depends on the presence of SMG7 and tethered SMG5 mutants that cannot interact with SMG7 are inactive in this assay [16]. Furthermore, the ability of SMG5 to induce degradation appears to be generally limited by cellular SMG7 levels and depends on SMG7 that can interact with UPF1 [28]. Similarly, Cho and colleagues showed that depletion of the decapping enhancer PNRC2 impairs the ability of tethered SMG5 to induce degradation of the target mRNA reporter [68]. PNRC2 was shown to bind simultaneously to a phospho-residue on UPF1 and to DCP1A [69]. Together, these findings suggest a hierarchy in the SMG5/SMG7-mediated branch of NMD in which SMG7 would mediate both i) the deadenylation of the mRNP by recruiting CCR4-NOT and ii) the coordination of SMG5 in the complex, before SMG5 subsequently aids in the coordination of the decapping and 5’-3’ exonucleolytic decay factors with UPF1.

### Concluding Remark

In conclusion, this work adds to the NMD toolbox BioID as a new and valuable tool for identifying even physically weak and transient interactions among proteins that would not persist under conditions used in immunoprecipitation protocols. The data presented here shows that besides confirming already well established interactions, many new putative interactors were indeed detected. Verification of these BioID hits remains however challenging. Although *in situ* proximity ligation assays can be used as a first independent approach to assess whether the biotinylated proteins indeed co-localize with the bait inside cells, only complementing functional data will in the future provide compelling evidence for an involvement of these proteins in NMD.

## Materials and Methods

### Molecular cloning

The humanized coding sequences for BirA and the BirA* were used [19, 70] and expression plasmids pcDNA3/H16 for HA-Gly_16_-tagged bait factors, pBioCTRL/HB and pBioID/HB* for HA-hBirA and HA-hBirA(R118G) bait fusions were generated by standard molecular cloning procedures. A detailed description of the expression constructs is provided in S1 File and sequences are available upon request.

### Cell culture and transfection

The 293T cells and HeLa Tet-Off TCRβ ter68 (clone 2.2) used in this study are described elsewhere [24]. They were cultured at 37°C under a 5% carbon dioxide atmosphere in DMEM-F12 (1:1) (Gibco) supplemented with 100 U/mL Penicillin/ 100 μg/mL Streptomycin (Gibco), 10% fetal calf serum (GeneOn), and additives as indicated.

Transfections for NMD depletion and restoration in HeLa TetR TCRβ ter68 (clone 2.2) cells were performed using Lipofectamine 2000 (Invitrogen). Amounts of plasmids needed for exogenous protein expression at near endogenous levels have been titrated. The day after transfection, cells were selected for shRNA expression plasmids carrying the puromycin resistance gene by 1.0 μg/mL puromycin dihydrochloride (Santa Cruz Biotechnology) over two days. Then, cells were exposed to 50 μM biotin (Sigma Aldrich) to boost biotinylation for 16 hours before harvesting.

For the set-up experiments in Fig 1 and for large-scale protein expressions in Figs 3–8, the 293T cells were transfected using Lipofectamine 2000 and expression was allowed for 2 days. Within the last day, cells were exposed to 50 μM biotin for 0, 6, and 16 hours before harvesting as indicated in the results.

For accumulation of hyperphosphorylated proteins such as UPF1, 293T cells were exposed to the phosphatase 2 (PP2) inhibitor okadaic acid at 50 μM for three hours before lysis.

### RNA analysis

Total RNA was extracted from cells using self-made Trizol [71]. After 2-propanol precipitation and two washes in 75% ethanol, the RNA was resuspended in 1 mM tri-sodium citrate, pH 6.5. Integrity was regularly assessed on agarose gels. One microgram total RNA was reverse transcribed by AffinityScript Multi-Temperature RT blend (Agilent Technologies) according to the manufacturer's recommendations with random hexamers (Microsynth). qRT-PCR assays contained the oligonucleotides described in S1 File, in either Brilliant III Ultra Fast QPCR Master Mix for TaqMan^®^ assays or Brilliant III Ultra Fast SYBR^®^ Green QPCR Master Mix. Pipetting was assisted by a CAS1200 robot (Corbett Life Science) and real-time fluorescence was recorded on a Rotorgene 6200 (Corbett Life Science). Data were analyzed using the ΔΔCT approach [72].

### Protein extracts

Detached cells were lysed by gentle hypotonic lysis in GHL solution (10 mM TRIS-HCl 10 mM sodium chloride, 2 mM EDTA, 0.5% Triton X-100, pH 7.8) supplemented with HALT protease inhibitor (Pierce), Phosphatase inhibitor tablets (Pierce), and 50 μM okadaic acid (Santa Cruz Biotechnology) for 20 min on ice. Subsequently, salt concentrations were adjusted to 150 mM sodium chloride and the crude lysates were cleared by centrifugation for 15 min at 10,000 g and 4°C. If indicated in the figure legends, 800 μg RNase A (Sigma Aldrich) was supplemented per equivalent of 10^7^ cells.

### Streptavidin affinity capture

50 μL Streptavidin magnetic beads (Pierce) per 10^7^ cell equivalents were used in the purification. First the beads were blocked in Odyssey Blocking Solution (TBS) (LI-COR) and washed in lysis buffer before addition to protein extracts. Input fractions for the affinity purification were collected before the beads were resuspended in cleared lysates equivalent of 1–2 10^7^ cells. For the tandem purification, the beads were resuspended in the acidic eluates from anti-HA immunoprecipitations (see below). Binding of biotinylated proteins was allowed for 1–16 hours at 4°C. Supernatants were then collected for the unbound fraction, and the beads were washed twice with 1.98% (w/v) lithium dodecyl sulfate, once with wash buffer 2 (50 mM HEPES-NaOH, 500 mM sodium chloride, 1 mM EDTA, 1% Triton X-100, 0.1% deoxycholate, pH 7.5), once with wash buffer 3 (10 mM TRIS-HCl, 250 mM lithium chloride, 1 mM EDTA, 0.5% Igepal CA-630, 0.5% deoxycholate, pH 8.1), and twice with buffer 4 (50 mM TRIS-HCl, 50 mM sodium chloride, pH 7.4), all at 4°C. The SA-bound proteins were eluted by equilibration in PAGE loading solution supplemented with 2–5 mM biotin followed by boiling at 95°C for 15 min (adapted from [73]).

### Hemagglutinin (HA) antibody immunoprecipitations

15 μL anti-HA magnetic beads (Pierce) per 10^7^ cell equivalents were used for immunoprecipitations. First the beads were blocked in Odyssey Blocking Solution (TBS) (LI-COR) and washed in TBS-0.05% NP40 buffer (50 mM TRIS-HCl pH 7.5, 150 mM sodium chloride, 0.05% IGEPAL CA-630) before addition to lysates. Beads were then resuspended in lysates equivalent of 1–2×10^7^ cells and binding was allowed for one hour at 4°C. The beads were then washed twice in TBST and once in ultrapure water. Immunoprecipitates were released from the beads in elution solution (2–5 mg/mL HA peptide, 0.1 M glycine pH 2.0) for 10 min at room temperature. Finally, the eluates were neutralized by addition of 1 M TRIS-HCl, pH 8.5. If required, PAGE loading buffer (Novex NuPAGE) was added and samples were boiled at 85°C for 10 min.

### Liquid chromatography–tandem mass spectrometry (LC–MS/MS)

Each lane was divided into 6 slices which were reduced, alkylated, trypsinized and peptides were desalted as previously described by [74]. After being dried in a speed-vac, the peptides were resuspended in 8 μL of solvent A (5% acetonitrile, 0.1% formic acid). LC-ESI-MS/MS of 5 μL of each sample was performed on a Fourier-transformed LTQ mass spectrometer (FT-LTQ, Thermo Electron, San Jose, CA). Peptides were separated on an Agilent chromatographic separation system 1100 (Agilent Technologies, Waldbronn, Germany) connected to a 15 cm fused-silica emitter of 75 μm inner diameter (New Objective, Inc. Woburn, MA USA), packed in-house with ReproSil-Pur C18-AQ 3 μm beads (Dr. Maisch Gmbh, Ammerbuch, Germany) with LC-gradient and MS method parameters previously described in [75].

### LC-MS/MS data analysis

Raw MS files were converted into peaklist (‘.msm’ files) via Raw2msm version 1.10_2007.06.14. All MS/MS samples were analyzed using Mascot (Matrix Science, London, UK; version 2.3.02) with the following parameters: Database UniProt_CP_Human_20150204 (89796 sequences; 35686673 residues), Taxonomy Homo sapiens, enzyme Trypsin, Maximum missed cleavages 2, fixed modification carbamidomethyl (C), variable modification oxidation (M), acetyl (protein n-terminus) and biotin (K), peptide tolerance 10 ppm, MS/MS tolerance 0.5 Da. Scaffold (version Scaffold_4.3.4, Proteome Software Inc., Portland, OR) was used to validate MS/MS based peptide and protein identifications. Peptides identification threshold was set at 95.0% probability by the Peptide Prophet algorithm [76] with Scaffold delta-mass correction, while protein identifications were accepted with a threshold of 99.0% applying the Protein Prophet algorithm [77] and contained at least 2 exclusive unique identified peptides. Proteins that contained similar peptides and could not be differentiated based on the MS/MS analysis alone were grouped to satisfy the principles of parsimony while the ones sharing significant peptide evidence were grouped into clusters. All the proteomic data as raw files and scaffold results (.sf3 format) were loaded on Peptide Atlas repository (accession number http://www.peptideatlas.org/PASS/PASS00768). Gene ontology (GO) analyses were performed with the list of identified proteins using the online implementation of the EnrichNet tools [78]. The enrichment of GO biological processes and the enrichment of InterPro annotations was calculated in Figs 3B, 6A and 6C, respectively. The analysis of the protein-protein interaction (PPI) network was performed with the PPI spider tool [34] using the experimental binary interactions deposited in the IntAct database. The interaction network was visualized in Cytoscape 3.2.1 [79] and graphically edited in Adobe Illustrator CS6.

### Western blotting and streptavidin probing

Proteins were resolved on self-made TRIS-glycine polyacrylamide gels or commercial Bis-Tris 4–12% NuPAGE gels (MOPS running buffer, Invitrogen). Proteins were transferred onto reinforced nitrocellulose (Optitran BA-S 85, GE Healthcare Life Sciences) in Bjerrum’s transfer buffer (48 mM TRIS, 39 mM glycine, 0.1% sodium dodecyl sulfate, 10% methanol, pH 9.2) by semi-dry electroblotting or by the iBlot protocol P3 (Invitrogen) with two modifications: i) The membrane was incubated in equilibration solution (2× NuPAGE transfer buffer, 10% (v/v) methanol, 0.1% (v/v) NuPAGE antioxidans) for 20–30 min before blotting, and ii) transfer time was increased to 8.5 min to allow blotting of larger proteins. For the dot blots in Fig 1, proteins from cleared lysates were absorbed onto nitrocellulose membranes using a Bio-Dot unit (Bio-Rad).

Before probing with streptavidin IRDye 680LT or IRDye 800CW (LICOR) (at 1/10,000 dilution), membranes were blocked in Odyssey Blocking Solution (TBS) (LICOR) containing 1% lithium dodecyl sulfate. The membrane was then washed five times in TBST (20 mM TRIS-HCl, pH 7.4, 150 mM sodium chloride, 0.1% Tween 20) and air-dried.

For normal western blotting, membranes were blocked with 5% low-fat milk in TBST before exposure to primary antibody at concentrations indicated in S1 File for 2–16 hours. After five washes in TBST, secondary antibodies fluorescently labeled with IRDye 680LT or IRDye 800CW (LICOR) were applied for 1.5–2 hours. Finally, membranes were washed five times in TBST and air-dried. Fluorescent signals were detected using an Odyssey scanner system (LICOR).

For detection of phosphorylated proteins, phospho blocking solution (6% (w/v) fat-free bovin serum albumin (Sigma Aldrich), 10 mM glycerol-2-phosphate, 0.1% (w/v) sodium azide, in TBST) was used for blocking membranes and for exposure to antibodies that bind phospho-residues. Also, TBST for the washes was supplemented with 10 mM glycerol-2-phosphate.

### Proximity ligation assays

The proximity ligation assays in Fig 8 were performed with the DuoLink^®^ system (Sigma Aldrich). HeLa TetR TCRβ ter68 (clone 2.2) were grown in LabTek^®^ Chamber Slides and, where indicated, were exposed to 33 μg/mL puromycin dihydrochloride (Santa Cruz Biotechnology) or to 100 ng/mL cycloheximide (Focus Biomolecules) for six hours, or to 150 μM sodium arsenite (Sigma Aldrich) for two hours. After washing with PBS, the cells were fixated in 4% paraformaldehyde in PBS for 20 min at 22°C. Fixation was stopped by five washes in IF buffer (2 mM magnesium chloride, 10% (v/v) glycerol, in TBS) and one wash in IF storage buffer (0.2 M glycine, 2 mM magnesium chloride, 0.1% sodium azide, in TBS). The cells were permeabilized in Perm/Block Solution (6% fat-free bovine serum albumin (Sigma Aldrich), 0.5% (v/v) Triton X-100, in TBS) for one hour. After washing with 0.1% Triton X-100 in TBS, primary antibodies were applied in TBS supplemented with 6% fat-free bovine serum albumin (Sigma Aldrich) and 0.1% (v/v) Triton X-100 and incubated over night at 4°C. Application of secondary antibodies, proximity ligation and signal amplification was performed according to the manufacturer’s protocol. The slides were mounted in Mowiol 4–88 (Sigma-Aldrich) and sealed with nail polish. Fluorescence microscopic images were taken on a Leica DMI6000 B with a Leica DFC360 FX monochrome digital camera. The primary images were analyzed with the LAS AF software (Leica Biosystems).

## Supporting Information

S1 File Supplementary Methods. Molecular cloning of constructs, oligonucleotides for qRT-PCR, and antibodies.
(DOCX)
