# Supplementary material for: Identification of Interactions in the NMD Complex Using Proximity-Dependent Biotinylation (BioID)
Source: PLoS One. 2016 Mar 2;11(3):e0150239. doi: 10.1371/journal.pone.0150239 (PMC4774922; doi:10.1371/journal.pone.0150239)
Supplement: S1 File — Molecular cloning of constructs, oligonucleotides for qRT-PCR, and antibodies. (DOCX) [file pone.0150239.s001.docx]

**Supplementary Methods**

**Molecular cloning**

PCR reactions were carried out with Herculase II Fusion DNA polymerase (Agilent Technologies) and all oligonucleotides were obtained from Microsynth. The coding sequence for the humanized BirA and the BirA(R118G) were obtained from [[80](#_ENREF_1), [81](#_ENREF_2)], PCR amplified using the primers 5’-AAT TCA CTC GAG ATG AAG GAC AAC ACC GTG CCC-3’ and 5’-AAT TCA TCT AGA CTA CTT CTC TGC GCT TCT CAG GG-3’, digested with XhoI and XbaI, and inserted into pcDNA3.1(+) whose multiple cloning site was adapted by insertion of a oligonucleotide hybrid of 5’-CTA GTT CTA GAC TCG AGG GAT CCG CTA GCA TGC ATT GAT CAG CGG CCG CA-3’ and 5’-CTA GTG CGG CCG CTG ATC AAT GCA TGC TAG CGG ATC CCT CGA GTC TAG AA-3’ in sense orientation into the NheI and XbaI sites. This resulted in the expression vector for the free HA-BirA and HA-BirA(R118G), pBioCTRL/HB and pBioID/HB*, respectively. They contain a BamHI and NotI site downstream of the HA-BirA coding sequence that was used to insert the RNAi-resistant coding sequence of the NMD factors UPF1, UPF2, and SMG5 in frame to the HA-BirA.

The UPF1 isoform 2 open-reading frame was amplified by PCR using the primers 5’-AAC CAA GGA TCC ATG AGC GTG GAG GCG TAC G-3’ and 5’-TAG ATG CGG CCG CTT AAT ACT GGG ACA GCC CCG-3’. The amplified product was digested with NotI and BamHI and ligated into the vectors pBioCTRL/HB and pBioID/HB* that was prepared accordingly. The plasmids are called pBioCTRL/HB0/UPF1^R^ and pBioID/HB*0/UPF1^R^, respectively.

The UPF2 open-reading frame was amplified by PCR using primers 5’-ACA CTA GGA TCC ATG CCA GCT GAG CGT AAA AAG C-3’ and 5’-ATA TAT GCG GCC GCT TAA CGT CTC CTC CCA CCA GTC-3’. The amplified product was digested with NotI and BamHI and ligated into the vectors pBioCTRL/HB and pBioID/HB* that was prepared accordingly. A small deletion originating from the template was then corrected by QuikChange Site-Directed Mutagenesis (Life Technologies) according to the manual. The final plasmids are called pBioCTRL/HB0/UPF2^R^ and pBioID/HB*0/UPF2^R^, respectively.

The SMG5 open-reading frame was assembled by fusion of three PCR products. They were generated using the primers 5’-GGA TAG GAT CCA TGA GCC AAG GCC CCC CCA CAG G-3’ and 5’-GTA CTG CTT GCT TCC TGC TCT CTC CAA G-3’, 5’-AGA GCA GGA AGC AAG CAG TAC AGT G-3' and 5’-ATG CCA CTC GGG TCC TCC TCA CCT G-3’, 5’-AGG TGA GGA GGA CCC GAG TGG CAT GGT GAC-3’ and 5’-TCA TTA TGC GGC CGC TCA ACC AAT TTC CTT CCA C-3’, respectively. They were pooled and used as template in the final PCR using the flacking primers 5’-GGA TAG GAT CCA TGA GCC AAG GCC CCC CCA CAG G-3’ and 5’-TCA TTA TGC GGC CGC TCA ACC AAT TTC CTT CCA C-3’ to fuse the complete coding sequence. The amplified product was digested with NotI and BamHI and ligated into the vectors pBioCTRL/HB and pBioID/HB* that was prepared accordingly. The plasmids are called pBioCTRL/HB0/SMG5^R^ and pBioID/HB*0/SMG5^R^, respectively.

The HA-Gly_16_ fusion proteins were derived from the pBioID/HB* fusion contructs by excision of the BirA(R118G) coding sequence with XhoI and BamHI and replacement with the oligonucleotide hybrid of 5’-TCG AGG GCG GAG GGG GCG GAG GAG GGG GCG GCG GAG GCG GCG GCG GAG GGG-3’ and 5’-GAT CCC CCT CCG CCG CCG CCT CCG CCG CCC CCT CCT CCG CCC CCT CCG CCC-3’. The resulting plasmids were named pcDNA3/H16/UPF1^R^, pcDNA3/H16/UPF2^R^, and pcDNA3/H16/SMG5^R^, respectively.

The pSUPuro plasmids that were used for RNA interference are described in Metze and colleagues [[82](#_ENREF_3)] with the exception of the pSUPuro UPF2 for which a novel target sequence was chosen (UPF2 target 3). Briefly, the deoxyoligonucleotides 5’-GAT CCC CGC ATT AAT TCA GAT GGC TTA GTT CAA GAG ACT AAG CCA TCT GAA TTA ATG CTT TTT GG AAA-3’ and 5’-AGC TTT TCC AAA AAG CAT TAA TTC AGA TGG CTT AGT CTC TTG AAC TAA GCC ATC TGA ATT AAT GCG GG-3’ were phosphorylated at the 5’-ends with polynucleotide kinase (New England Biolabs) in T4 ligation buffer (Fermentas), hybridized with each other, and ligated into the BglII and HindIII sites of pSUPuro.

**RNA interference**

The shRNA target sequences for the pSUPuro plasmids are as follows: pSUPuro scrambled, 5’-ATT CTC CGA ACG TGT CAC G-3’; pSUPuro UPF1 target 2, 5’-GAG AAT CGC CTA CTT CAC T-3’; pSUPuro UPF1 target 4, 5’-GAT GCA GTT CCG CTC CAT T-3’; pSUPuro UPF2 target 3, 5’-GCA TTA ATT CAG ATG GCT TAG-3’; pSUPuro SMG5, 5’-GAA GGA AAT TGG TTG ATA C-3’.

**Antibodies**

The antibodies used in this study are listed in the S2 Table. The minimal amount of cell extracts for successful detection and the corresponding antibody dilution are listed. For western blots in this study, generally 5×10^5^ cell equivalents of cell extract in input and unbound fractions were resolved by SDS-PAGE.

The rabbit polyclonal UPF1 antibody (UPF1, batch 2) that was used in Figure 3A was generated as follows: Briefly, a cDNA fragment, amplified by PCR, encoding for amino acids 1061-1118 of human UPF1 (isoform 2) was cloned into pET28a. The recombinant protein was expressed in *Escherichia coli* BL21 (DE3) Codon Plus RIPL and purified under denaturing conditions over Ni-NTA beads (Qiagen) according to the manufacturer’s instructions. The purified protein was dialyzed against PBS and rabbits were immunized with the purified protein in combination with GERBU Adjuvant LQ. UPF1 antibodies were then affinity purified from the serum over a synthetic peptide (corresponding to UPF1 isoform 2 residues 1061-1086) coupled via an n-terminal cysteine to a SulfoLink immobilization column (ThermoFisher Scientific) according to the manufacturers instruction.

**S1 Table. Assays for real-time qRT-PCR.**

| **Gene Name** | **Assay Type** | **Reference Transcripts** | **Amplicon Region** | **Oligonucleotide Sequences,**  **Fluorophores** | **References** |
| --- | --- | --- | --- | --- | --- |
| *NMD factors* | | | | | |
| UPF1 | TaqMan® | NM_002911.3 | exon 3 | 5’-TGCAACGGACGTGGAAATAC-3’  5’-ACCTCTTTGCATTTTGCCCTC-3’  5’-FAM-TCTGGCAGCCACATTGTAAATCACCTTG-BHQ1-3’ | [[83](#_ENREF_4)] |
| UPF2 | SYBR® Green | NM_015542.3,  NM_080599.2 | 3’-UTR | 5’-AGCAGCACGTGTCATTTC-3’  5’-TGTGTCCACTGCTCTCATTC-3’ | [[84](#_ENREF_5)] |
| SMG5 | TaqMan® | NM_015327.1 | exon 16 / exon 17 | 5’-GTCAGCATTGCCCAGTCTGA-3’  5’-AGCCTGTTCCGACGAGCTT-3’  5’-FAM-AGGCACAGTTCCGAATGGCACA-BHQ1-3’ | [[83](#_ENREF_4)] |
| *TCRβ reporter and normalisers* | | | | | |
| TCRβ | TaqMan® | minigene derived from mouse TRB, NG_001333 | exon VDJβ / exon C_1_ | 5’-GCGGTGCAGAAACGCTGTA-3’  5’-TGGCTCAAACAAGGAGACCTT-3’  5’-FAM-CTCGAGGATCTGAGAAATGTGACTCCACC-TAMRA-3’ | [[85](#_ENREF_6)] |
| TCRβ | SYBR® Green | minigene derived from mouse TRB, NG_001333 | exon VDJβ / exon C_1_ | 5’-AGTTGGCTTCCCTTTCTCAG-3’  5’-CTTGGGTGGAGTCACATTTC-3’ | this study |
| ACTB | TaqMan® | NM_001101.3 | exon 5 / exon 6 | 5’-CTGGCACCCAGCACAATG-3’  5’-GCCGATCCACACGGAGTACT-3’  5’-FAM-ATCAAGATCATTGCTCCTCCTGAGCGC-BHQ1-3’ | [[82](#_ENREF_3)] |
| ACTB | SYBR® Green | NM_001101.3 | exon 5 / exon 6 | 5’-TCCATCATGAAGTGTGACGT-3’  5’-TACTCCTGCTTGCTGATCCAC-3’ | [[84](#_ENREF_5)] |

**S2 Table. Antibodies used in this study.**

| **Factor** | **Antibody** | **Source** | **Article_number** | **Dilution** | **Minimal cell**  **equivalents** |
| --- | --- | --- | --- | --- | --- |
| UPF1,  batch 2 | rabbit pAb IgG | home-made | none | 2,000 | 2×10^5^ ceq |
| UPF1,  RENT1 | goat pAb IgG | Bethyl Laboratories | A300-038A | 1,000 | 2×10^5^ ceq |
| UPF2 | rabbit pAb IgG | Jens Lykke-Andersen | none | 3,000 | 3×10^5^ ceq |
| UPF2,  RENT2 | rabbit pAb IgG | Bethyl Laboratories | A303-929A | 1,000 | 3×10^5^ ceq |
| UPF3B,  Rent3 (H-190) | rabbit pAb IgG | Santa Cruz Biotechnology | sc-48800 | 500 | 3×10^5^ ceq |
| SMG1 | rabbit pAb IgG | Bethyl Laboratories | A301-535A | 1,000 | 3×10^5^ ceq |
| SMG5 | rabbit pAb IgG | Abcam | ab33033 | 1,000 | 3×10^5^ ceq |
| SMG6 | rabbit pAb IgG | Abcam | ab87539 | 1,000 | 2×10^5^ ceq |
| SMG7 | rabbit pAb IgG | Bethyl Laboratories | A302-170A | 1,000 | 5×10^5^ ceq |
| phospho-UPF1,  phospho-(S/T)Q, ATM/ATR/SMG1 substrates | rabbit pAb IgG | Cell Signaling Technology | 2851L | 1,000 | 3×10^5^ ceq |
| EIF4A3 | rabbit pAb IgG | Melissa Moore | none | 1,000 | 3×10^5^ ceq |
| RBM8A,  Y14 | rabbit pAb IgG | Silvia Barabino | none | 1,000 | 3×10^5^ ceq |
| CASC3  BTZ | rabbit pAb IgG | Bethyl Laboratories | A302-472A | 5,000 | 3×10^5^ceq |
| PABPC1 | mouse mAb IgG1 | Sigma Aldrich | P6246-200UL | 1,000 | 2×10^5^ ceq |
| ALYREF  ALY (11G5) | mouse mAb IgG1 | Santa Cruz Biotechnology | sc-32311 | 500 | 5×10^5^ ceq |
| RPS2 | mouse mAb IgG2a | Abcam | ab58341 | 250 | 5×10^5^ ceq |
| EIF4A2 | mouse mAb IgG2b | Santa Cruz Biotechnology | sc-137147 | 2,000 | 3×10^5^ ceq |
| EIF4A2 | rabbit pAb IgG | Abcam | ab31218 | 1,000 | 3×10^5^ ceq |
| CRKL  CRK-L (C-20) | rabbit pAb IgG | Santa Cruz Biotechnology | sc-319 | 1,000 | 3×10^5^ ceq |
| DCP1A | rabbit pAb IgG | Bethyl Laboratories | A303-590A | 1,000 | 3×10^5^ ceq |
| DCP2 | rabbit pAb IgG | Bethyl Laboratories | A302-597A | 1,000 | 3×10^5^ ceq |
| EDC4 | rabbit pAb IgG | Jens Lykke-Andersen | none | 2,000 | 5×10^5^ ceq |
| DDX6 | goat pAb IgG | Santa Cruz Biotechnology | sc-51415 | 500 | 5×10^5^ ceq |
| XRN1 | rabbit pAb IgG | Bethyl Laboratories | A300-443A | 1,000 | 3×10^5^ ceq |
| CNOT7,  hCAF1 | rabbit pAb IgG | Bertrand Séraphin | none | 1,000 | 3×10^5^ ceq |

**Supplementary References**

80. Mechold U, Gilbert C, Ogryzko V. Codon optimization of the BirA enzyme gene leads to higher expression and an improved efficiency of biotinylation of target proteins in mammalian cells. J Biotechnol. 2005;116(3):245-9. Epub 2005/02/15. doi: S0168-1656(04)00608-X [pii] 10.1016/j.jbiotec.2004.12.003. PubMed PMID: 15707685.

81. Roux KJ, Kim DI, Raida M, Burke B. A promiscuous biotin ligase fusion protein identifies proximal and interacting proteins in mammalian cells. J Cell Biol. 2012;196(6):801-10. Epub 2012/03/14. doi: jcb.201112098 [pii] 10.1083/jcb.201112098. PubMed PMID: 22412018; PubMed Central PMCID: PMC3308701.

82. Metze S, Herzog VA, Ruepp MD, Muhlemann O. Comparison of EJC-enhanced and EJC-independent NMD in human cells reveals two partially redundant degradation pathways. RNA. 2013;19(10):1432-48. doi: 10.1261/rna.038893.113. PubMed PMID: 23962664; PubMed Central PMCID: PMCPMC3854533.

83. Yepiskoposyan H, Aeschimann F, Nilsson D, Okoniewski M, Muhlemann O. Autoregulation of the nonsense-mediated mRNA decay pathway in human cells. RNA. 2011;17(12):2108-18. doi: 10.1261/rna.030247.111. PubMed PMID: 22028362; PubMed Central PMCID: PMCPMC3222124.

84. Balistreri G, Horvath P, Schweingruber C, Zund D, McInerney G, Merits A, et al. The host nonsense-mediated mRNA decay pathway restricts Mammalian RNA virus replication. Cell Host Microbe. 2014;16(3):403-11. Epub 2014/09/12. doi: S1931-3128(14)00299-6 [pii] 10.1016/j.chom.2014.08.007. PubMed PMID: 25211080.

85. Buhler M, Wilkinson MF, Muhlemann O. Intranuclear degradation of nonsense codon-containing mRNA. EMBO Rep. 2002;3(7):646-51. doi: 10.1093/embo-reports/kvf129. PubMed PMID: 12101097; PubMed Central PMCID: PMCPMC1084183.
